# The Use of an Amino Acid Formula Containing Synbiotics in Infants with Cow’s Milk Protein Allergy—Effect on Clinical Outcomes

**DOI:** 10.3390/nu13072205

**Published:** 2021-06-27

**Authors:** Katy Sorensen, Abbie L. Cawood, Lisa H. Cooke, Dionisio Acosta-Mena, Rebecca J. Stratton

**Affiliations:** 1 Medical Affairs, Nutricia Ltd., White Horse Business Park, Trowbridge BA14 0XQ, UK; 2 Institute of Human Nutrition, Faculty of Medicine, Mailpoint 113, Southampton General Hospital, Tremona Road, Southampton SO16 6YD, UK; a.l.cawood@soton.ac.uk (A.L.C.); r.j.stratton@soton.ac.uk (R.J.S.); 3 Department of Nutrition and Dietetics, Bristol Royal Hospital for Children, Upper Maudlin Street, Bristol BS2 8BJ, UK; lisa.cooke@uhbw.nhs.uk; 4 Cegedim Health Data, Cegedim Rx, London SW8 3QJ, UK; dionisio.acostamena@cegedimrx.co.uk

**Keywords:** paediatrics, dietetics, cow’s milk protein allergy, synbiotics, amino acid formula, infections

## Abstract

Cow’s milk protein allergy (CMPA) is common and costly. Clinical trials of infants with CMPA have shown that the use of an amino acid formula containing pre- and probiotics (synbiotics) (AAF-Syn) may lead to significant reductions in infections, medication prescriptions and hospital admissions, compared to AAF without synbiotics. These effects have not yet been confirmed in real-world practice. This retrospective matched cohort study examined clinical and healthcare data from The Health Improvement Network database, from 148 infants with CMPA (54% male, mean age at diagnosis 4.69 months), prescribed either AAF-Syn (probiotic *Bifidobacterium breve* M16-V and prebiotics, including chicory-derived oligo-fructose and long-chain inulin) or AAF. AAF-Syn was associated with fewer symptoms (−37%, *p* < 0.001), infections (−35%, *p* < 0.001), medication prescriptions (−19%, *p* < 0.001) and healthcare contacts (−18%, *p* = 0.15) vs. AAF. Infants prescribed AAF-Syn had a significantly higher probability of achieving asymptomatic management without hypoallergenic formula (HAF) (adjusted HR 3.70, 95% CI 1.97–6.95, *p* < 0.001), with a shorter clinical course of symptoms (median time to asymptomatic management without HAF 1.35 years vs. 1.95 years). AAF-Syn was associated with potential cost-savings of £452.18 per infant over the clinical course of symptoms. These findings may be attributable to the effect of the specific synbiotic on the gut microbiome. Further research is warranted to explore this. This real-world study provides evidence consistent with clinical trials that AAF-Syn may produce clinical and healthcare benefits with potential economic impact.

## 1. Introduction

Cow’s milk protein allergy (CMPA) is a hypersensitive immune reaction to cow’s milk protein (CMP) [1], which typically develops within the first few months of life and is thought to affect around 2%–5% of infants in Europe [1,2,3,4,5]. Children with CMPA may present with gastrointestinal (GI), skin and/or respiratory symptoms, including anaphylaxis in some severe cases [1]. Additionally, 3.7% of children with food allergies may present with faltering growth [6]. Management requires the avoidance of CMP, and breastfeeding remains the best option, with maternal exclusion diets required in some but not all cases [1]. Formula-fed infants may require hypoallergenic formulae (HAF) to help manage symptoms, with extensively hydrolysed formulae (eHF) indicated first-line and amino-acid formulae (AAF) recommended for more complex cases, such as in the presence of anaphylaxis, faltering growth or other severe symptoms, or where symptoms do not resolve with eHF [1,4,7,8].

Whilst CMPA may be managed in primary care, referral to other allergy specialists, including a paediatric Dietitian, may be considered [9]. Across the UK, management of CMPA and its symptoms in the first year alone is estimated to account for over 336,000 general practice visits, 12,000 outpatient visits and 1200 hospital admissions, amounting to approximately £25.6 million in healthcare costs per year [10]. Most children outgrow their CMPA within 3–4 years of diagnosis, with 51% acquiring tolerance to CMP within 2 years [11,12,13]. Time to a tolerance of CMP may be affected by several factors, including immunoglobulin-E (IgE) status, sensitivity to other allergens, and family history of progression to atopic conditions such as asthma, rhinitis or eczema [1]. Research suggests that gut dysbiosis may also impact the clinical course of CMPA [14,15].

The infant gut microbiome, which is thought to begin colonising *in utero*, plays an important role in immune function by influencing development, modulating responses and promoting intestinal barrier processes [14,15]. Dysbiosis of the gut microbiome may disrupt mucosal immunological tolerance and trigger pro-allergic and inflammatory processes, which, in turn, may lead to food allergies and other atopic conditions [14]. In light of this, the microbiome is a potential therapeutic target for managing allergic disease [14]. Studies have demonstrated clinical benefits of modulating the infant gut microbiota with pre- or probiotics when given within HAF or as a supplement to formulae, including a reduction in atopic dermatitis (AD) and an earlier resolution of CMPA [16,17,18,19]. In particular, randomised controlled trials (RCTs) have shown that infections, hospital admissions and the use of antibiotics and concurrent medications occurred in fewer infants who were fed a specific AAF containing both pre- and probiotics (synbiotics) compared to those who received an AAF without synbiotics [20,21,22,23,24]. However, real-world evidence (data collected by clinicians as part of the routine care of patients) investigating these effects in the clinical setting is lacking. The aim of this study was to compare clinical symptoms, infections, healthcare usage and clinical course, among infants with CMPA prescribed an AAF containing synbiotics or an AAF without pre- or probiotics. Potential economic implications were also considered.

## 2. Materials and Methods

### 2.1. Study Design

We conducted a retrospective cohort study that aimed to compare case records extracted from The Health Improvement Network (THIN) database (A Cegedim Proprietary Database) of infants with CMPA managed with an amino-acid formula containing synbiotics (AAF-Syn) or a standard amino-acid formula without pre- or probiotics (AAF).

### 2.2. The Health Improvement Network (THIN) Database

In 2020, the THIN database contained longitudinal data from 2.9 million anonymised active patient records from approximately 365 practices, which is generalisable to the UK population [25]. These records include patients’ demographics, diagnoses, clinical symptoms, healthcare professional contacts, referrals, procedures and prescriptions issued by the GP from their entire medical history, providing a comprehensive picture of actual clinical practice in the UK. Clinical data is recorded within the THIN database using read-codes, a coded thesaurus of clinical terms which has been used by UK healthcare professionals since 1985 [26]. Prescription data is recorded using Anatomical Therapeutic Chemical (ATC) codes, an index of unique codes maintained by the World Health Organisation, which are assigned to medicines according to their mechanism, or their target organ or system [27]. The THIN database has been cited in the methodologies of over 1000 research publications to date and offers a unique insight into real-world clinical practice in the UK [28].

### 2.3. Study Population

**Figure 1:**
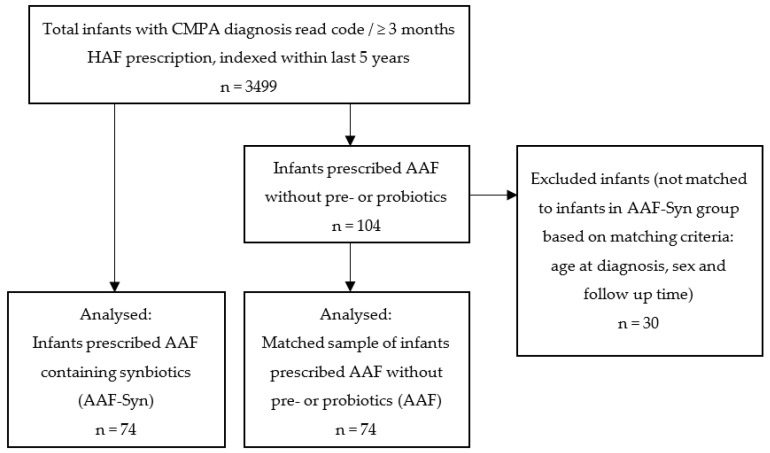
Flowchart summarising the study population selection. Abbreviations: CMPA: cow’s milk protein allergy; HAF: hypoallergenic formulae; AAF: amino acid formula; AAF-Syn: amino acid formula containing synbiotics.

At the point of data extraction (4 November 2020), the THIN database contained anonymised case records indexed within the last 5 years of 3499 infants with confirmed or suspected CMPA at ≤12 months of age. Confirmed CMPA was defined by a CMPA diagnosis read code. In the absence of a CMPA read-code, suspected CMPA was defined by the prescription of a HAF for at least 3 consecutive months (88% eHF; 35% AAF). Of the 3499 infants with confirmed or suspected CMPA, all infants who had been prescribed an AAF supplemented with the probiotic *Bifidobacterium breve* M16-V and prebiotics (including chicory-derived oligo-fructose and long-chain inulin) (AAF-Syn group, n = 74) were included in the study. An additional 104 infants had been prescribed an AAF without pre- or probiotics. From these, a sample of 74 infants was selected by one-to-one matching with infants from the AAF-Syn group, matched for age at diagnosis, sex and observation period (mean 1.19 years), for inclusion in the study (AAF group) (Figure 1). Formula switches were expected in the case records. To account for these, designation of infants to each group was determined by their exposure to the AAF-Syn or AAF, respectively, as a proportion of the total time that they had been prescribed HAF, ensuring that no infants in the AAF-Syn group had greater exposure to the AAF than to the AAF-Syn, and vice versa.

Infants were excluded from the dataset if they had a history of diagnosed intestinal failure; necrotising enterocolitis; cancer, malignancy or tumour; congenital heart disease; cystic fibrosis; cerebral palsy; metabolic conditions; chromosomal anomalies; or if they were prescribed any other medical nutrition product not indicated for CMPA.

### 2.4. Study Variables and Outcome Measures

The following data were extracted for baseline characteristics and outcomes from all infants’ case records:

- Baseline (birth to diagnosis) characteristics. Due to limited detail within read-codes regarding symptom severity, this included several proxies for disease severity (presenting symptoms, number of organ systems affected, history of infections, other allergies, faltering growth, eHF prescription and the mean number of allergic medication prescriptions).
- Clinical symptoms. This included all-cause symptoms (symptoms documented in case records related to any cause) and allergic symptoms, including GI, skin and respiratory symptoms. Examples of symptoms classified as GI symptoms included GI illness, diarrhoea, constipation, flatulence, vomiting, reflux, bloody stools, mucus in stools and colic. Examples of skin symptoms included eczema and urticaria, and respiratory symptoms included asthma and rhinitis. GI, skin and respiratory symptoms were also aggregated into overall allergic symptoms.
- Infections. This included all-cause infections and infection sub-categories (GI, skin, respiratory and ear infections). GI, skin, respiratory and ear infections were also aggregated into overall allergic symptoms.
- Healthcare usage. This included all-cause prescriptions, prescriptions of antibiotics, dermatological medications, anti-reflux medications, all-cause healthcare contacts, Dietitian contacts, hospital admissions and specialist referrals. Prescriptions of antibiotics, dermatological medications and anti-reflux medications were aggregated into overall medication prescriptions, and Dietitian contacts, hospital admissions and specialist referrals were aggregated into overall healthcare contacts.

The combined persistence of allergic symptoms (and those of other affected systems related to CMPA) and an ongoing HAF prescription was considered as an indicator of the ongoing burden of allergic disease and its wider impact on the general health of the infant and on the healthcare system. A consecutive period of at least 3 months of no symptoms and no HAF prescription was used as a composite outcome to comparatively estimate the clinical course of symptoms requiring HAF for each cohort.

All outcomes were measured from CMPA diagnosis over the duration of available data for each infant (referred to as the observation period throughout, mean 1.19 years for both cohorts). Results were presented for the individual outcomes and the overall result of aggregated outcomes, as the proportion (%) of infants who had the outcome at least once during their observation period. Due to variability in the observation period between infants in the cohort, results were also presented as the individual outcome rate per person-year. All-cause outcomes were presented as a rate per person-year.

Person-years were calculated by multiplying the number of infants in each cohort by the cumulative time (years) that the infants in the cohort were followed. Person-year rates were calculated by dividing the total number of events for a specific outcome that occurred in the cohort by the number of person-years for the cohort.

All statistical analyses were performed using R software, version 4.0.2 [29]. Statistical significance was set at *p* < 0.05. Differences in proportional data between groups were measured using either Fisher’s exact test or chi-square test of independence, where appropriate. Differences in rates between groups were measured with the Poisson test.

A Cox proportional hazards (PH) regression model was used to determine unadjusted and adjusted hazard ratios (HRs) for AAF-Syn compared to AAF for the composite outcome of at least 3 months of no symptoms and no HAF prescription. This model was adjusted for relevant confounders, specifically: sex, age at first symptoms, age at first HAF prescription and time between diagnosis and first HAF prescription. Combined symptoms and HAF persistence probability (survival) curves were generated from the model to compare probability distributions of symptom persistence and ongoing HAF prescription between groups, and the median time to asymptomatic management without HAF was estimated for each group. Sensitivity analysis was undertaken by calculating the relative difference in time to asymptomatic management without HAF from the adjusted HRs, using methods described elsewhere [30].

An indicative cost analysis was used to compare the cost of healthcare usage between the two groups over the observed clinical course of symptoms, similar to methods described elsewhere [31]. Individual costs (Table 1) included prescriptions (AAF-Syn, AAF, antibiotics, dermatological medication and anti-reflux medications), Dietitian contacts and hospital admissions.

The costs for prescribing the relevant AAF-Syn or AAF were calculated using prices listed on the Monthly Index of Medical Specialties [32], based on the estimated mean intakes calculated from prescription dosage and duration data within the THIN database. The costs for medication prescriptions were obtained from the England Prescription Cost Analysis [33], based on the most commonly used medications in the cohort and assuming the most conservative costs from the listed national ingredient costs per item.

Individual costs for Dietitian appointments and other specialist referrals were obtained from Unit Costs of Health and Social Care 2020 [34]. The latter was based on referral data within the THIN database, conservatively based on the type of paediatric allergy specialist with the lowest unit cost, and assumed that each referral led to one appointment and no follow up. Hospital admission costs were obtained from the 2020/21 National Tariff Payment System [35]. The reason for hospital admission was not available in the dataset; therefore, the method calculated a mean paediatric admission costs from the range [35], based on the most common type of infection among the cohort (Table 1). Sensitivity analysis was also undertaken using the minimum and maximum average paediatric admission costs.

Healthcare usage rates per person-year were extrapolated to the respective median time to asymptomatic management without HAF for each group to estimate average costs per infant over the observed clinical course of symptoms. For example, the cost of antibiotic prescriptions would be calculated using the unit cost (£0.83) (Table 1) multiplied by the antibiotic prescription rate per person-year and the median clinical course of symptoms for each cohort.

## 3. Results

### 3.1. Demographics

The infants’ characteristics are presented in Table 2. No significant demographic differences were found between groups at baseline.

### 3.2. Management of CMPA

The mean ages at which infants first presented with symptoms associated with CMPA (0.56 months vs. 0.48 months, *p* = 0.2) and at which they were diagnosed with CMPA or received their first HAF prescription (4.71 months vs. 4.67 months, *p* > 0.9) were similar between the AAF-Syn group and the AAF group (Table 2). All infants had used at least one other HAF prior to the AAF-Syn or AAF, with nearly equal proportions of infants in each group having been prescribed an eHF previously (72% AAF-Syn; 73% AAF). Of infants in the AAF-Syn group, 61% had been prescribed the AAF at some point in their clinical history, with a mean prescription duration for the AAF of 1.7 months (median 0 months). None of the infants in the AAF group had been prescribed the AAF-Syn.

Overall, the AAF-Syn group was prescribed HAF for less time than those on AAF (10.59 months vs. 13.69 months), though this was not statistically significant. Their length of time on AAF-Syn (6.65 months) was significantly shorter than the AAF group’s time on AAF (8.44 months) (Table 2). The proportion of total time on HAF for which the AAF-Syn group was prescribed the AAF-Syn (63%) and for which the AAF group was prescribed the AAF (62%) was similar. Over the observation period, the mean prescription of HAF powder (calculated from the estimated total quantity prescribed, divided by the duration of the prescription) was 148 g/day for AAF-Syn and 134 g/day for AAF.

### 3.3. Disease Severity

There were no significant differences between groups in any proxies for disease severity recorded in the database at baseline. Presenting symptoms, the number of systems affected, infections, other allergies, faltering growth, eHF prescription and the mean number of prescriptions for the management of allergic symptoms were similar between groups (Table 2).

### 3.4. Outcome Measures

#### 3.4.1. All-Cause Symptoms, Infections and Healthcare Usage

Compared to AAF, AAF-Syn was associated with significantly lower rates of all-cause clinical symptoms (−37%), infections (−35%) and medication prescriptions (−19%) (Table 3). The rate of all-cause healthcare contacts was also lower among infants in the AAF-Syn group per person-year (−18%), although this was not significant.

#### 3.4.2. Allergic Symptoms

During the observation period, significantly fewer infants in the AAF-Syn group had overall allergic (GI, skin and/or respiratory) symptoms than in the AAF group (32% vs. 61% overall, *p* < 0.001). A breakdown of allergic symptoms is shown in Table 4. Significantly fewer infants experienced GI symptoms (−50%) and skin symptoms (−58%) with AAF-Syn than with AAF. Similarly, AAF-Syn was associated with lower rates of GI and skin symptoms per person-year, which was statistically significant for GI symptoms. Recorded respiratory symptoms were low across the entire cohort, with no significant differences between groups.

Further analysis related to the clinical course of symptoms and HAF prescription over time is reported at the end of the results section.

#### 3.4.3. GI, Skin, Respiratory and Ear Infections

During the observation period, significantly fewer infants in the AAF-Syn group had overall GI, skin, respiratory and/or ear infections than in the AAF group (66% vs. 86% overall, *p* = 0.007). A breakdown of these infections is shown in Table 4. Respiratory infections were the most recorded type of infection among infants in both groups. Compared to the AAF group, significantly fewer infants in the AAF-Syn group had respiratory infections during the observation period (−30%), with a significantly lower rate of respiratory infections per person-year (−32%). Skin infections affected 28% fewer infants in the AAF-Syn group compared to the AAF group during the observation period, with a 41% lower rate per person-year, although this was not statistically significant. Whilst the proportion of infants experiencing GI infections was similar between groups, the rate of GI infections per person-year was significantly lower among the AAF-Syn group. There were no differences between groups in the proportion of infants with ear infections or the rate of ear infections per person-year.

#### 3.4.4. Healthcare Usage

##### Antibiotic, Dermatological and Anti-Reflux Medications

During the observation period, fewer infants in the AAF-Syn group overall were prescribed antibiotic, dermatological, and/or anti-reflux medications compared to the AAF group, though not significantly so (84% vs. 95% overall, *p* = 0.064). While the proportion of infants prescribed antibiotics was similar between groups (AAF-Syn 41% vs. AAF 57%, *p* = 0.070), the rate of antibiotic prescriptions was significantly lower (−47%) with AAF-Syn than with AAF (0.883 vs. 1.664 per person-year, *p* < 0.001). Similarly, whilst the between-group difference in the proportion of infants prescribed dermatological medications was not significant (AAF-Syn 54% vs. AAF 68%, *p* = 0.13), the rate of dermatological medication prescriptions was significantly lower (−18%) with AAF-Syn than with AAF (3.056 vs. 3.735 per person-year, *p* = 0.016). Differences between the AAF-Syn and AAF groups were not statistically significant for the percentage of infants prescribed anti-reflux medications (49% vs. 57%, *p* = 0.4) or the rate of anti-reflux medication prescriptions (2.784 vs. 2.411 per person-year, *p* = 0.135).

##### Dietitian Contacts, Hospital Admissions and Specialist Referrals

During the observation period, there were no statistically significant differences between groups in the proportion of infants who had overall Dietitian contacts, hospital admissions and/or specialist referrals (AAF-Syn 53% vs. AAF 58% overall, *p* = 0.6). Whilst the number of infants who had Dietitian contacts was similar between groups (AAF-Syn 31% vs. AAF 35%, *p* = 0.7), the rate of Dietitian contacts was 44% lower with AAF-Syn than with AAF (0.328 vs. 0.588 per person-year, *p* = 0.014). There were no significant differences in the proportion of infants who were admitted to the hospital (AAF-Syn 36% vs. AAF 42%, *p* = 0.6) or in the rates of hospital admissions per person-year between groups (0.826 vs. 0.781, *p* = 0.801). Between-group differences were not significant for specialist referrals when considering proportion of infants (AAF-Syn 1.4% vs. AAF 4.1%, *p* = 0.6) or rates per person-year (AAF-Syn 0.011 vs. AAF 0.057, *p* = 0.219).

#### 3.4.5. Clinical Course of Symptoms and HAF Prescription

**Figure 2:**
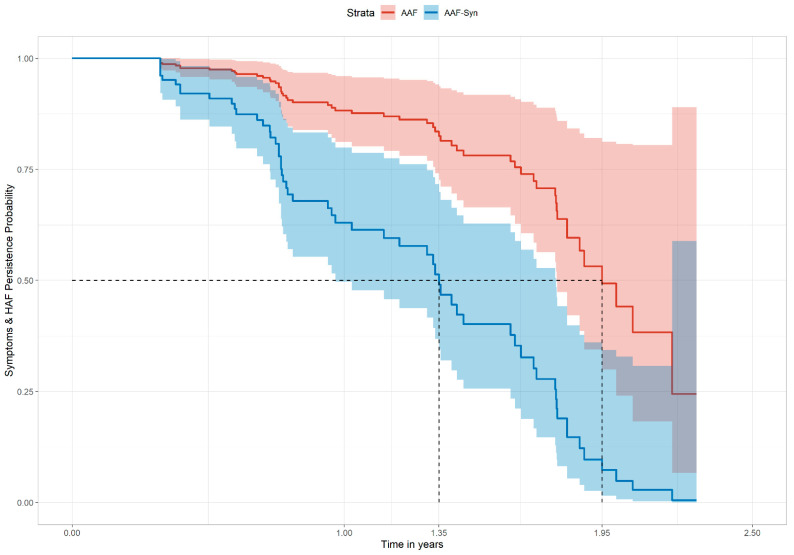
The adjusted Cox proportional hazard regression model showing the combined probability of symptom persistence and ongoing HAF prescription over time. Abbreviations: HAF: hypoallergenic formulae; AAF: amino acid formula; AAF-Syn: amino acid formula with synbiotics.

The Cox PH regression model showed that the probability distribution of persistent symptoms and ongoing HAF prescription was significantly different between groups. In the matched cohort, infants receiving AAF-Syn had a significantly higher probability of achieving at least 3 months of no symptoms and no HAF prescription (adjusted HR 3.70, 95% CI 1.97–6.95, *p* < 0.001; unadjusted HR 3.28 (95% CI 1.88–5.74), *p* < 0.001). For infants receiving AAF-Syn, this was associated with a shorter median time to asymptomatic management without HAF compared to those receiving AAF (1.35 years vs. 1.95 years) (Figure 2). Probability of symptom persistence and ongoing HAF prescription was also lower among infants receiving AAF-Syn than AAF at 12 months (0.61 (95% CI 0.48–0.79) vs. 0.88 (95% CI 0.80–0.96)) and 18 months (0.40 (95% CI 0.26–0.63) vs. 0.78 (95% CI 0.66–0.92)).

#### 3.4.6. Indicative Cost-Analysis

An indicative cost analysis was undertaken for each group using standard healthcare costs (Table 1), based on the prescription data during the observation period for AAF-Syn (148 g/day for 6.65 months (75 tins at £24.82 per tin) = £1861.50) and AAF (134 g/day for 8.44 months (86 tins at £22.98 per tin) = £1976.28) and the extrapolated medication and healthcare rates over the clinical course of symptoms. Compared to AAF, AAF-Syn was associated with lower healthcare costs, equating to differences of £14.36 for medication prescriptions, £64.75 for Dietitian appointments, £22.82 for other specialist referrals and £235.46 for hospital admissions per infant.

Overall, this generated a potential mean cost-saving associated with AAF-Syn of £452.18 per infant over the clinical course of symptoms. With an infant population size of 745,263 in the UK [36], assuming CMPA prevalence of 2.5% (conservative estimate from the 2%–5% range reported elsewhere [1,2,3,4,5]), and assuming that 35% of infants with CMPA may be prescribed an AAF, extrapolating these savings to a simple budget impact model generates a potential saving of £2,948,659.08 across the UK over this time period.

## 4. Discussion

To our knowledge, this is the first study from real-world data comparing the clinical outcomes of infants with CMPA who are managed with AAF containing synbiotics (*Bifidobacterium breve* M16-V and prebiotics, including chicory-derived oligo-fructose and long-chain inulin) or an AAF without pre- or probiotics. These findings suggest that AAF-Syn may be more effective in managing allergic symptoms compared to AAF, with 48% fewer infants experiencing allergic symptoms and a 37% reduction in symptom rate per person-year. These are important findings for infants and their families, as allergic symptom severity has been reported to be significantly correlated with poor quality of life (QoL) [37]. Moreover, 23% fewer infants experienced overall GI, skin or respiratory infections with AAF-Syn, with an additional 35% reduction in infection rate per person-year. Research has also demonstrated that recurrent infections may negatively affect the QoL of both children and caregivers [38,39].

The impact of these findings extends beyond infants and their families, with wider implications for the healthcare system. HAF is estimated to account for up to 38% of the healthcare costs of managing CMPA in the first year after diagnosis [10]. There may be direct potential cost savings arising from the observed earlier discontinuation of AAF-Syn compared to AAF found in the current study. Furthermore, the 19% reduction in rates of medication prescriptions and 18% reduction in rates of healthcare usage observed with AAF-Syn were associated with additional potential cost-savings, particularly when accounting for the clinical course over which symptoms persisted.

The clinical course of symptoms and HAF prescription among the AAF group (median 1.95 years) was consistent with the time of resolution for around half of the children with CMPA, at around 2 years, which has been reported elsewhere [1] and is seen generally in clinical practice. Interestingly, data from this study shows that AAF-Syn was associated with a 31% shorter median clinical course of symptoms when compared to AAF, equating to an approximate difference of 7.2 months. Sensitivity analysis found similar results when calculated from the unadjusted and adjusted HRs, respectively, as described in the methods (27%–30% shorter clinical course).

Whilst it is not possible to determine whether this result corresponds to complete resolution of CMPA in all cases, this notable finding, that the use of AAF-Syn is associated with a shorter clinical course of symptoms when compared to an AAF without synbiotics, is an important consideration to factor into any cost-analysis. Our indicative cost-analysis, which accounted for this, found that AAF-Syn was associated with potential cost savings (£452.18 per infant) due to lower overall prescription costs and less use of health care resources over the period that the infants experienced symptoms. This cost analysis was undertaken using the average cost of paediatric hospital admission for respiratory symptoms, as the most common type of infection reported among the cohort and a relatively conservative cost within the range of paediatric admission types. Sensitivity analysis using the minimum and maximum costs for paediatric hospital admission for respiratory infections found that potential cost savings could range from £390.86 to £539.32 with AAF-Syn.

The findings of this study are consistent with clinical trials of the only AAF-Syn currently available on prescription in the UK, which have shown significant reductions in infections, medications and hospital admissions when compared to AAF in infants with CMPA [20,21,22,23,24]. It is possible that the benefits seen are related to the clinical effect of the specific synbiotic, which may have modified the dysbiotic gut microbiome of infants with CMPA, bringing it closer to that observed in studies of healthy breastfed infants [20,21,22,23,40,41]. The precise mechanism of the synbiotic effect remains unknown; however, the improvement of the gut microbiota profile through synbiotics may inhibit the growth of pathogenic organisms, thereby helping to reduce the incidence of infections [42] and associated healthcare usage in turn. Although it is not possible to attribute causation from observational methods, considering the consistency of findings from the current study and wider literature, the constituent-specific effects of this AAF-Syn formulation may have contributed to the reductions in allergic symptoms, infections and healthcare usage, and therefore, in turn, to the associated potential cost savings observed in this study. It would be interesting to investigate whether the effects of AAF-Syn bring the clinical experiences of infants with CMPA more in line with those of non-allergic infants. Further research is warranted to explore this and the impact of other interventions, such as early dietetic input, which may also improve clinical outcomes and reduce associated costs.

Whilst this retrospective analysis of case records from the THIN database offers novel findings from clinical practice in real-world settings, to add to the growing evidence base of a specific AAF-Syn from RCTs [20,21,22,23,24], the applicability of the findings to eHF containing synbiotics (eHF-Syn) is unknown. Evidence from RCTs suggests that eHF supplemented with the same synbiotic blend may be associated with lower usage of medications in infants with atopic dermatitis and with greater improvement in skin symptoms among infants with an IgE-mediated response [43,44]. Other studies using THIN data have also compared CMPA management with eHF or AAF [45] and with eHF or eHF containing probiotics [46], however to date, no real-world studies of eHF-Syn have been undertaken and would warrant further research.

This study has some limitations. Firstly, evidence from the THIN database relies on clinicians recording information in their patients’ records with read-codes. This may not be performed consistently, such as for CMPA diagnosis read-codes, which were infrequently recorded in the dataset and lacked detail as to the type (IgE or non-IgE mediated) of CMPA. Variations in recording practices for symptoms and infections may also be present. For example, a GP may not document symptoms if a diagnosis can be made at the point of consultation, yet they may document the symptom of a suspected infection whilst awaiting confirmation of a correct diagnosis. The clinical differentiation of an allergic symptom and an infection may present further challenges, as each may have similar presentations. All-cause clinical and healthcare outcomes were therefore included to increase the sensitivity of observations. Due to the matching of cohorts, it is likely that any error introduced from this will apply equally to both cohorts. Furthermore, the significantly lower rate of antibiotic prescriptions among the AAF-Syn cohort is consistent with the significant reduction in recorded infections.

Additionally, the sample size of this cohort study may be considered a potential limitation. Nevertheless, the achievement of statistical significance for the key composite outcome, of at least 3 months of no symptoms and no HAF, suggests that the data provides sufficient evidence to support the observation that the use of AAF-Syn has an effect on this outcome. Furthermore, both unadjusted and adjusted HRs are large enough (>3) to suggest an observable effect in practice. Whilst the 95% CI for the adjusted HR is wider than it would be with a larger sample size, the lower bound is still sufficiently large (1.97) to provide evidence of a clear association even under a conservative interpretation of the results.

A further limitation related to the retrospective analysis of longitudinal records, which may introduce potential confounding factors, such as any differences in the severity of disease at baseline or exposure to other hypoallergenic formulas during the observation period. From the perspective of analysing the natural history of CMPA, switching HAF prescription is part of real-world clinical practice, and to exclude data of infants who had switched formulas would be to exclude the relevant clinical experiences of many infants. Consequently, we aimed to control for these potential confounders in several ways.

Firstly, groups were well matched at baseline, with a similar mean age of the first presentation to the GP with symptoms and of CMPA diagnosis and similar distributions of disease severity proxies. Therefore, it was assumed that the presentation and severity of CMPA were equally distributed between groups. To account for the potential confounding effect of formula switches within cohorts, the mean exposure time on either AAF-Syn or AAF (according to the assigned cohort) was calculated as a percentage of the overall time that the infant had been prescribed HAF, with no significant differences between groups. Indeed, whilst some of the AAF-Syn group had been prescribed the AAF at some point, their mean prescription duration on the AAF was only 1.7 months, and none of the AAF group had been prescribed the AAF-Syn. Furthermore, the Cox PH regression was adjusted for baseline differences in HAF variables. To note, baseline differences in the respective mean prescriptions of AAF-Syn and AAF were assumed to be reflective of the slight differences in the standard concentrations of the formulae and equate to similar daily amounts between groups (approximately 1028 mL (699 kcal, 19.5 g protein) for AAF-Syn and 971 mL (680 kcal, 18.4 g protein) for AAF) when constituted at the standard dilutions [47,48]. Differences may also have been apparent in other aspects of infants’ clinical management, including the type of medication, dietary compliance and previous exposure to other formulae or pre- or probiotics. Whilst we may try to control for some of these variables in the analysis, it is not possible to completely mitigate their potential effect on the results. Therefore, whilst it is not possible to ascertain causation from retrospective methodology, the differences observed in clinical outcomes are unlikely to be attributable to any baseline differences between cohorts but rather to their clinical management, including the choice of AAF. Further real-world evidence studies are warranted, with larger sample sizes and methodologies that could potentially allow the inclusion of time-dependent covariates into the Cox PH regression model to adjust for HAF switches and changes in HAF dosage [49,50] to advance our understanding of the effects of HAF containing synbiotics on clinical outcomes and associated healthcare costs.

## 5. Conclusions

CMPA presents a burden to infants, families and the healthcare system. This retrospective cohort study provides real-world evidence to suggest that infants with CMPA managed with AAF-Syn have a significantly shorter clinical course of symptoms requiring HAF and lower rates of symptoms, infections and healthcare, than those managed with AAF alone. These clinical benefits are associated with potential cost savings for healthcare services. Future studies with analytical methodologies which can further control for potential confounders are warranted.

## Figures and Tables

**Table 1:** A summary of cost estimates used in the indicative cost analysis.

Resource	Cost Per Infant Per Item
AAF-Syn (per 400 g tin) [32]	£24.82
AAF (per 400 g tin) [32]	£22.98
Antibiotics (amoxicillin) ^a^	£0.83
Dermatological (soft paraffin) ^a^	£3.76
Anti-reflux (ranitidine) ^a^	£0.83
Dietitian contact ^b^	£92
Other specialist (consultant paediatrician) contact ^c^	£237
Hospital admission ^d^	£577.33

AAF-Syn: amino acid formula with synbiotics (Neocate Syneo^®^, Nutricia, Liverpool, United Kingdom); AAF: amino acid formula (SMA^®^ Alfamino^®^, Nestle Health Science, Konolfingen, Switzerland); ^a^ Assuming the lowest cost from a range, “Individual Preparations” Section [33]; ^b^ Based on the unit cost for a Dietitian appointment (group session or one-to-one) [34]; ^c^ Based on the unit cost for an average paediatric consultant-led outpatient attendance, assuming referral led to one appointment [34]; ^d^ Calculated mean non-elective tariff for paediatric hospital admission for upper respiratory tract infections [35].

**Table 2:** Infants’ baseline characteristics.

Characteristic	AAF-Syn	AAF
Sex, n (%)		
Male	39 (53%)	40 (54%)
Female	35 (47%)	34 (46%)
IMD quintile, n (%)		
5th	27 (36%)	16 (22%)
4th	14 (19%)	18 (24%)
3rd	12 (16%)	11 (15%)
2nd	11 (15%)	9 (12%)
1st	10 (14%)	20 (27%)
Mean age at presentation of first symptoms associated with CMPA (months)	0.56 ± 0.30	0.48 ± 0.27
Mean age at CMPA diagnosis/first HAF prescription (months)	4.71 ± 2.48	4.67 ± 2.56
Mean duration on all HAF prescriptions (months)	10.59 ± 6.82	13.69 ± 9.36
Mean duration on the AAF-Syn/AAF (months)	6.65 ± 5.30 *	8.44 ± 5.62 *
Mean prescription of the AAF-Syn/AAF (g/day)	148 ± 78 *	134 ± 89 *
Proportion of infants with presenting symptoms before CMPA diagnosis, n (%)		
GI	31 (42%)	27 (36%)
Skin	17 (23%)	18 (24%)
Respiratory	0 (0%)	2 (2.7%)
Proportion of infants with multiple systems affected (GI/respiratory/skin), n (%)		
<2 systems affected	65 (88%)	67 (91%)
≥2 systems affected	9 (12%)	7 (10%)
Proportion of infants with infections before CMPA diagnosis, n (%)	32 (43%)	29 (39%)
Proportion of infants with other allergy ^†^, n (%)	9 (12%)	12 (16%)
Proportion of infants with faltering growth before CMPA diagnosis, n (%)	1 (1.4%)	2 (2.7%)
Proportion of infants with eHF prescription, n (%)	53 (72%)	54 (73%)
Mean number of allergic medication prescriptions ^‡^ before CMPA diagnosis	10.50 ± 7.61	10.68 ± 7.51
Mean observation period (years)	1.19 ± 0.57	1.19 ± 0.57

AAF-Syn: amino acid formula with synbiotics; AAF: amino acid formula; IMD: index of multiple deprivations; CMPA: cow’s milk protein allergy; HAF: hypoallergenic formula; GI: gastrointestinal; eHF: extensively hydrolysed formula; ± standard deviation; * *p* < 0.05; ^†^ Including read-codes documented in case-records for egg allergy, peanut allergy, food allergy, history of drug allergy, history of non-drug allergy and allergic reaction unspecified; ^‡^ Prescriptions of anti-reflux, dermatological and antibiotic medications documented in case-records.

**Table 3:** Rate per person-year of all-cause ^a^ symptoms, infections, medications and healthcare contacts with AAF-Syn vs. AAF.

Outcome	AAF-Syn (*n* = 74)	AAF (*n* = 74)	*p*-Value
Symptoms	2.43	3.88	<0.001
Infections	1.82	2.81	<0.001
Medication prescriptions	16.08	19.81	<0.001
Healthcare contacts	1.17	1.43	0.15

AAF-Syn: amino acid formula with synbiotics; AAF: amino acid formula; ^a^ Outcomes documented in case-records related to any and all causes.

**Table 4:** Incidence of symptoms and infections with AAF-Syn vs. AAF as the proportion of infants affected and rate per person-year.

		AAF-Syn (*n* = 74)	AAF (*n* = 74)	*p*-Value
Symptoms				
GI symptoms	Proportion	23%	46%	0.006
	Rate	0.43	0.72	0.013
Skin symptoms	Proportion	11%	26%	0.033
	Rate	0.17	0.32	0.066
Respiratory symptoms	Proportion	0%	2.7%	0.5
	Rate	0	0.02	0.5
Infections				
GI infections	Proportion	0	7%	0.058
	Rate	0	0.10	0.004
Skin infections	Proportion	26%	36%	0.2
	Rate	0.26	0.44	0.056
Respiratory infections	Proportion	59%	84%	0.002
	Rate	1.46	2.16	<0.001
Ear infections	Proportion	11%	11%	>0.9
	Rate	0.10	0.10	>0.9

AAF-Syn: amino acid formula with synbiotics; AAF: amino acid formula.

## Data Availability

The data that support the findings of this study are available from the corresponding author upon reasonable request.
